# Supplementary material for: Combinatory Treatment of Canavanine and Arginine Deprivation Efficiently Targets Human Glioblastoma Cells via Pleiotropic Mechanisms
Source: Cells. 2020 Sep 30;9(10):2217. doi: 10.3390/cells9102217 (PMC7600648; doi:10.3390/cells9102217)
Supplement: Supplementary file 1 [file cells-09-02217-s001.zip › Suppl figures Karatsai et al.docx]

Supplementary material

Combinatory Treatment of Canavanine and Arginine Deprivation Efficiently Targets Human Glioblastoma Cells via Pleiotropic Mechanisms

Olena Karatsai ^1^, Pavel Shliaha^2^, Ole N Jensen^2^, Oleh Stasyk ^3^ and Maria Jolanta Rędowicz ^1,^*

1. Nencki Institute of Experimental Biology, Polish Academy of Sciences, 3 Pasteur Str., 02-093 Warsaw, Poland.
2. Department of Biochemistry and Molecular Biology and VILLUM Center for Bioanalytical Sciences, University of Southern Denmark, DK-5230 Odense M, Denmark.
3. Department of Cell Signaling, Institute of Cell Biology, National Academy of Sciences of Ukraine, 14/16 Drahomanov Str., 79005 Lviv, Ukraine.

***** Correspondence: [m.redowicz@nencki.gov.pl](mailto:m.redowicz@nencki.gov.pl); Tel.: +48225892456

**Content:**

**Supplementary Information S1:** Experimental procedures: MS/MS method settings.

**Supplementary Figure S1**: Western Blot analysis of the apoptotic markers in U251MG and U87MG glioblastoma cells.

**Supplementary Figure S2**: Western Blot analysis of markers of apoptotic cell death in rat glia cells.

**Supplementary Figure S3**: Western Blot analysis of nuclear lamina proteins (lamins A/C and B1) in rat glia cells.

**Supplementary Figure S4**: Analysis of the focal adhesions in rat glia cells after 48 h of treatment with 50 µM canavanine under CM and AFM conditions.

**Supplementary Figure S5**: Western Blot analysis of proteins involved in cell adhesion in U251MG and U87MG cell lines.

**Supplementary Figure S6**: The level of Akt kinase in U251MG and U87MG cells.

**Supplementary Figure S7**: Gene otology analysis of proteins with canavanine incorporation against the set of all identified proteins.

**Supplementary Figure S8**: Illustration of spectrum cohesion for QLEDGRTLSDYNIQK peptide.

**Supplementary Figure S9**: Analysis of the ER stress marker, GRP78, in rat glia cells after 48 h of treatment with 50 µM canavanine under CM and AFM conditions.

**Supplementary Figure S10**: Effect of 50 µM canavanine on the ER in U251MG and U87MG glioblastoma cell lines.

**Supplementary Figure S11**: Canavanine (50 µM) evokes early glioblastoma cell stress response already after 4 h of treatment under arginine deprivation.

**Supplementary Figure S12**: Western Blot analysis of the markers of ER stress in rat glia cells treated with 50 µM canavanine under CM and AFM conditions.

**Supplementary Figure S13**: The level of the heat shock proteins HSP70 and HSP60 as a markers of mitochondrial stress.

**Supplementary Table S1a:** Arginine and canavanine sites.

**Supplementary** **Table S1b:** Peptides with Arginine and canavanine variants.

**Supplementary** **Table S2a:** Proteins used in GO analysis.

**Supplementary** **Table S2b:** GO analysis.

**Karatsai et al. Supplementary Information S1**

Experimental procedures: MS/MS method settings.

Orbitrap Fusion Lumos Method Summary

Global Settings

Use Ion Source Settings from Tune = False

Method Duration (min)= 100

Ion Source Type = NSI

Spray Voltage: Positive Ion (V) = 2800

Spray Voltage: Negative Ion (V) = 600

Sweep Gas (Arb) = 0

Ion Transfer Tube Temp (°C) = 275

APPI Lamp = Not in use

Pressure Mode = Standard

Default Charge State = 2

Experiment 1

Start Time (min) = 0

End Time (min) = 100

Cycle Time (sec) = 3

Scan MasterScan

MSn Level = 1

Use Wide Quad Isolation = False

Detector Type = Orbitrap

Orbitrap Resolution = 120K

Mass Range = Normal

Scan Range (m/z) = 375-1500

Maximum Injection Time (ms) = 50

AGC Target = 400000

Microscans = 1

RF Lens (%) = 30

Use ETD Internal Calibration = False

DataType = Profile

Polarity = Positive

Source Fragmentation = False

Scan Description =

Filter MIPS

MIPS Mode = Peptide

Filter ChargeState

Include undetermined charge states = False

Include charge state(s) = 2-7

Include charge states 25 and higher = False

Filter DynamicExclusion

Exclude after n times = 1

Exclusion duration (s) = 60

Mass Tolerance = ppm

Mass tolerance low = 10

Mass tolerance high = 10

Exclude isotopes = True

Perform dependent scan on single charge state per precursor only = False

Filter IntensityThreshold

Maximum Intensity = 1E+20

Use Signal Intensity Range = False

Minimum Intensity = 5000

Data Dependent Properties

Data Dependent Mode= Cycle Time

Scan Event 1

Scan ddMSnScan

MSn Level = 2

Isolation Mode = Quadrupole

Isolation Window = 2

Use Isolation m/z Offset = False

Multi-notch Isolation = False

Scan Range Mode = Auto Normal

FirstMass = 110

Scan Priority= 1

ActivationType = CID

Collision Energy (%) = 35

Activation Q = 0.25

Multistage Activation = False

Is EThcD Active = False

Detector Type = IonTrap

Ion Trap Scan Rate = Rapid

Maximum Injection Time (ms) = 300

AGC Target = 3000

Inject ions for all available parallelizable time = True

Microscans = 1

Use ETD Internal Calibration = False

DataType = Centroid

Polarity = Positive

Source Fragmentation = False**Karatsai et al. Supplementary Figure S1**


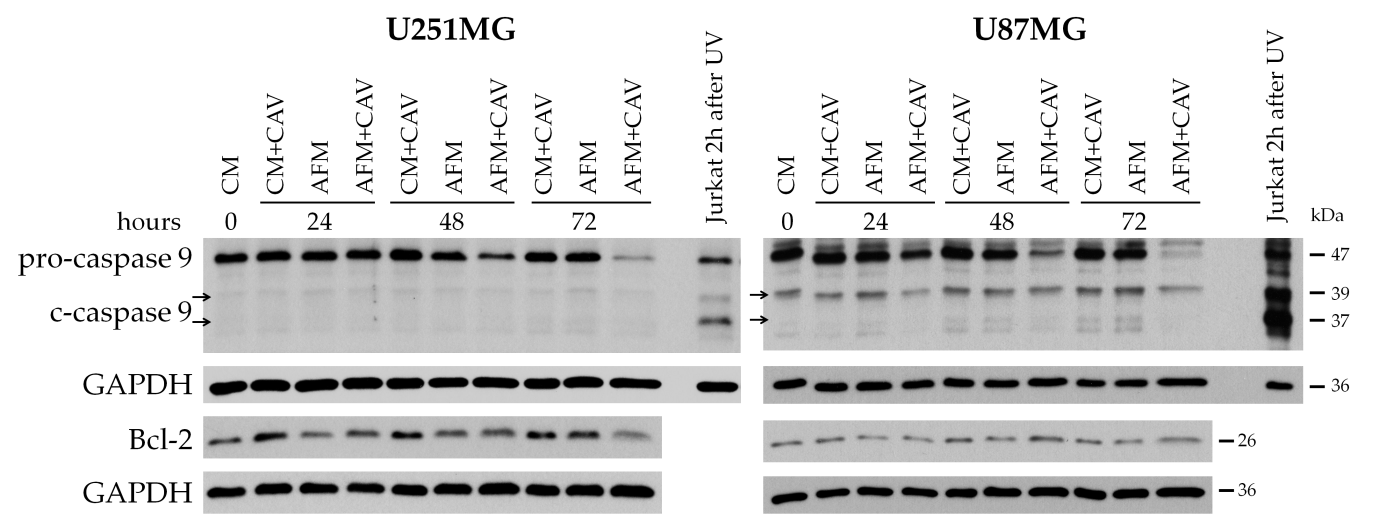


S**upplementary Figure S1**. Western Blot analysis of the apoptotic markers in U251MG and U87MG glioblastoma cells. Cells were cultivated under complete (CM) or arginine-free (AFM) conditions in combination with 100 µM canavanine (CAV) treatment. The levels of pro-caspase 9 and its cleaved form (c-caspase 9), and Bcl-2 up to 72 h of treatment were analyzed. GAPDH was used as a protein loading control. UV irradiated Jurkat cells were used as a positive control.

**Karatsai et al. Supplementary Figure S2**


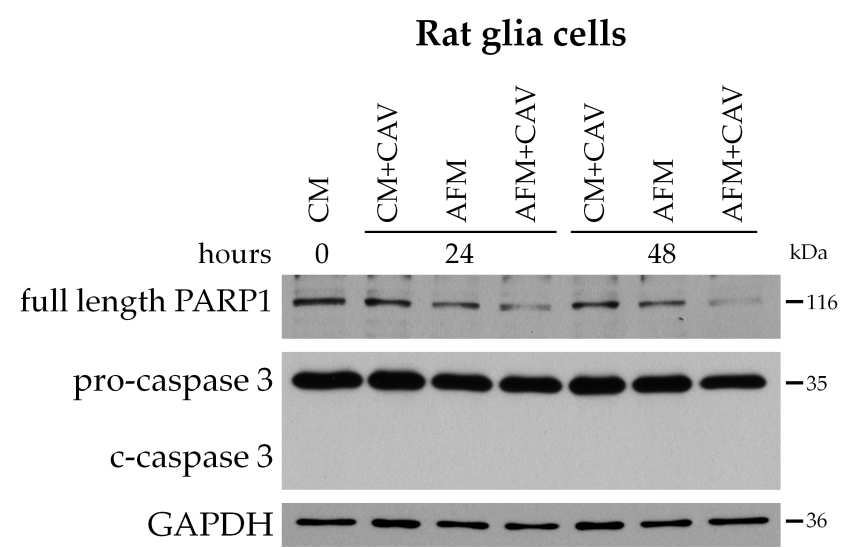


**Supplementary Figure S2.** Western Blot analysis of the markers of apoptotic cell death in rat glia cells. PARP1 and caspase 3 were detected in cell lysates after 100 µM canavanine treatment under CM and AFM conditions. No detection of cleaved form of PARP1 and caspase 3 (c-caspase 3). GAPDH was used as a protein loading control.

**Karatsai et al. Supplementary Figure S3**


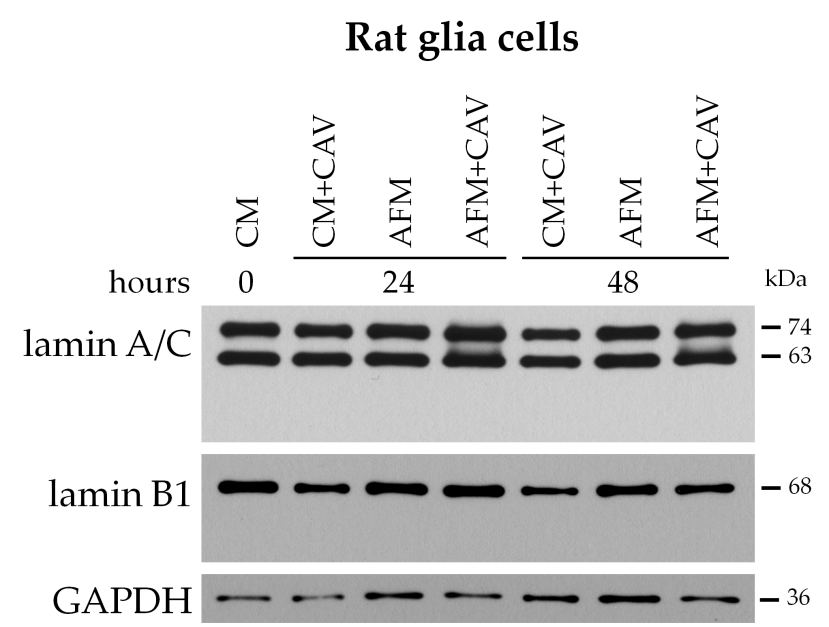


**Supplementary Figure S3.** Western Blot analysis of nuclear lamina proteins (lamins A/C and B1) in rat glia cells. GAPDH was used as a protein loading control. Cells were treated with 50 µM canavanine under CM and AFM conditions.

**Karatsai et al. Supplementary Figure S4**

**
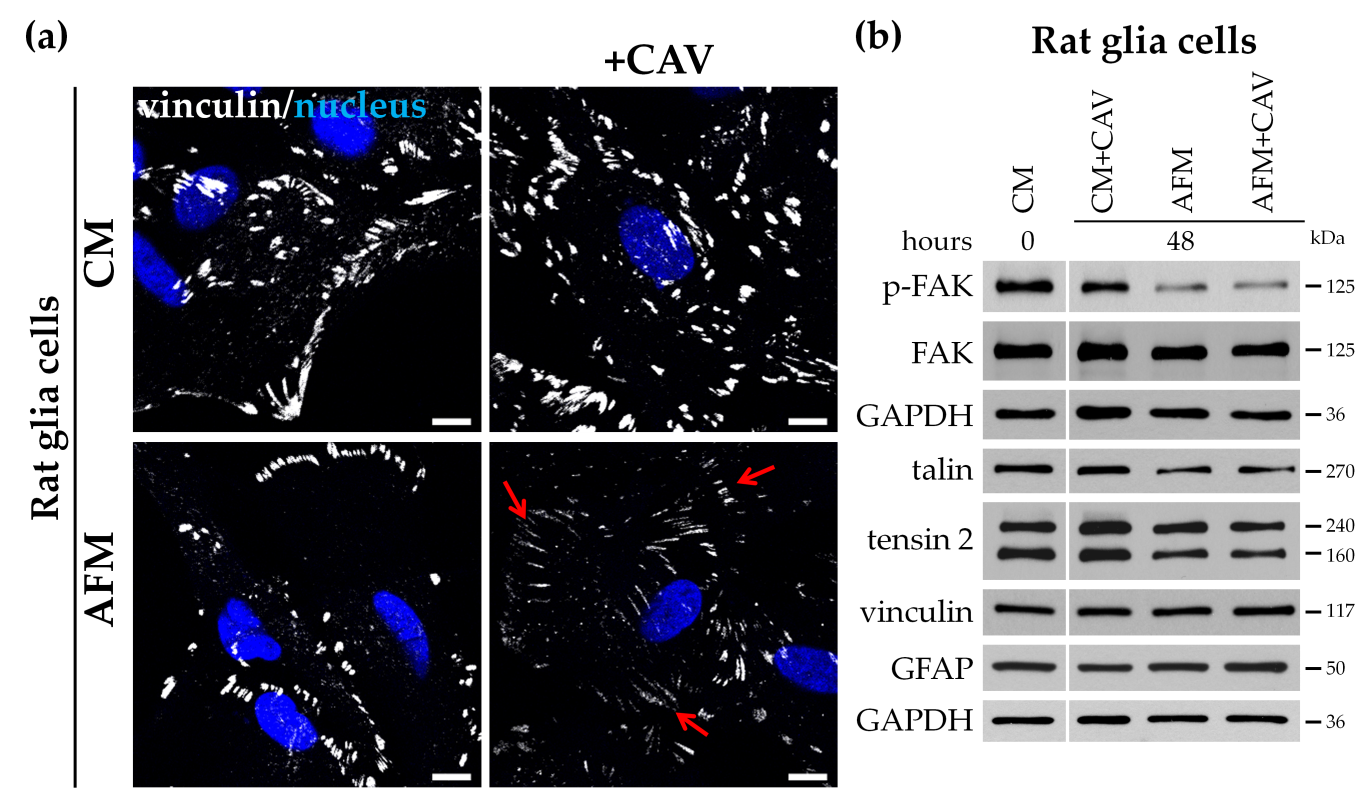
**

**Supplementary Figure S4.** Analysis of the focal adhesions in rat glia cells after 48 h of treatment with 50 µM canavanine under CM and AFM conditions. **(a)** Immunocytochemical staining for vinculin. Nuclei were labelled with DAPI. Bars, 10 µm. Arrows point to altered focal adhesion contacts. **(b)** Western Blot analysis of proteins involved in cell adhesion and motility. GFAP and GAPDH were used as a protein loading controls.

**Karatsai et al. Supplementary Figure S5**


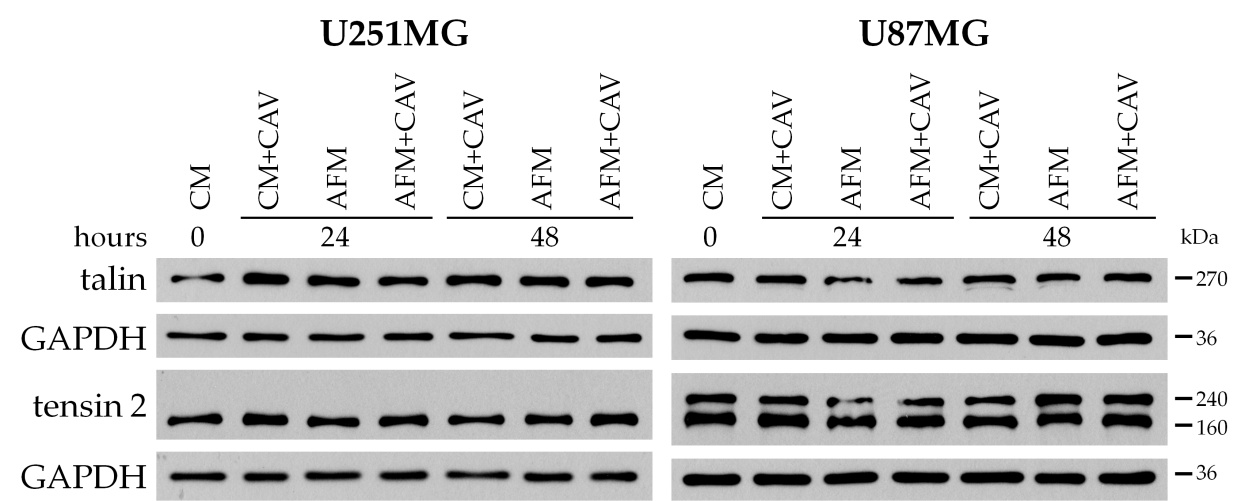


Supplementary Figure S5. Western Blot analysis of proteins involved in cell adhesion in U251MG and U87MG cell lines. Cells were treated with 50 µM canavanine under CM and AFM conditions. The analysis of cell lysates was made for talin and tensin 2. GAPDH was used as a protein loading control.

**Karatsai et al. Supplementary Figure S6**


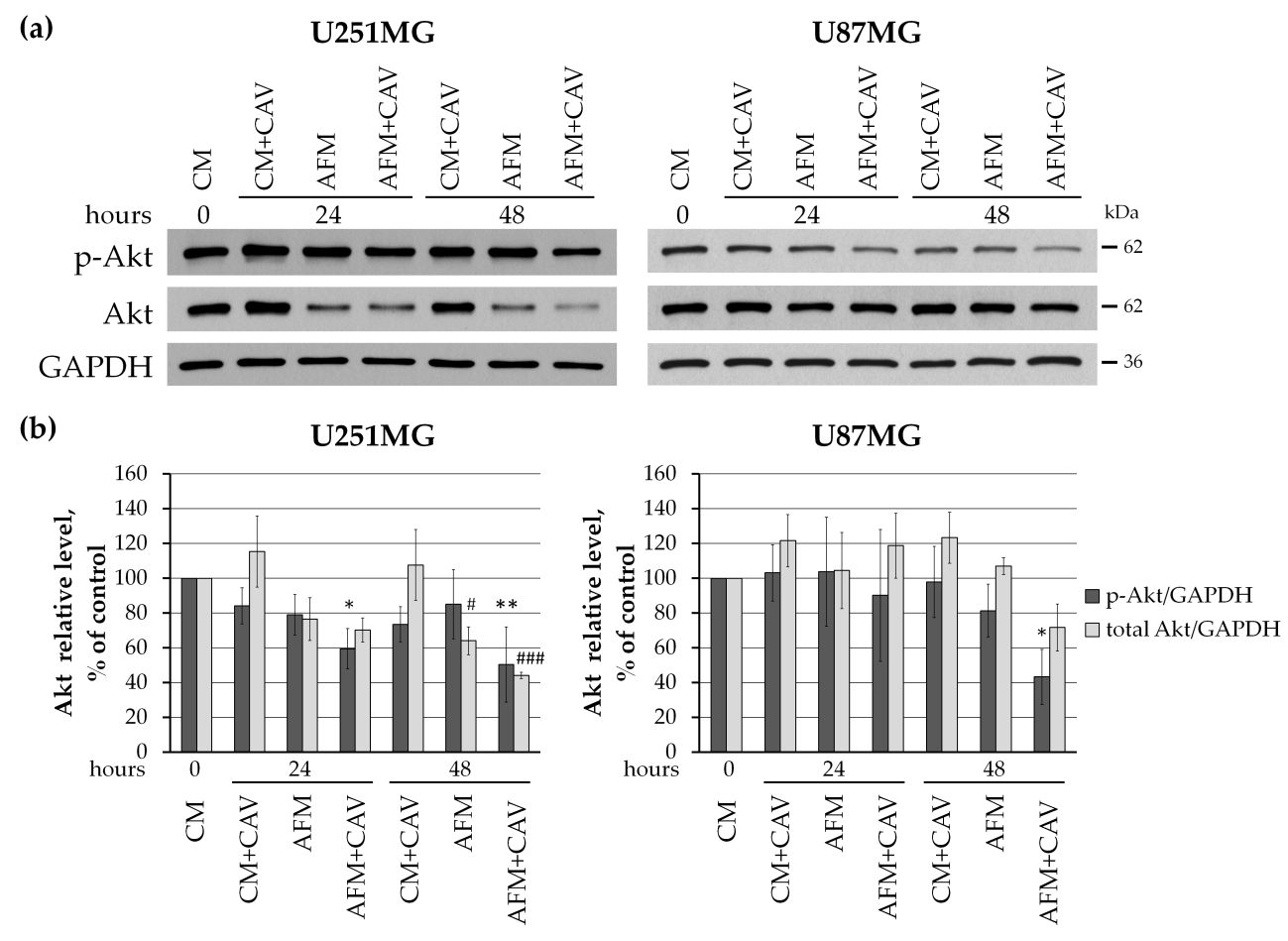


**Supplementary Figure S6.** The level of Akt kinase in U251MG and U87MG cells. **(a)** The level of Akt kinase and its phosphorylated (S473) active form were quantified by Western blot analysis. **(b)** The densitometry of the Akt level. GAPDH was used for protein loading control. Graph bars represent mean ±SD from three independent experiments. * p< 0.05, ** p<0.01 relative to p-Akt level in cells cultured in CM (control, 100%). # p<0.05, ## p<0.01 relative to total Akt level in cells cultured in CM (control, 100%).

**Karatsai et al. Supplementary Figure S7**

**Supplementary Figure S7.** Gene otology analysis of proteins with canavanine incorporation against the set of all identified proteins. **(a)** Biological process ontology, **(b)** cellular component ontology, **(c)** molecular function ontology, **(d)** top3 protein quantitation of proteins with and without canavanine incorporation.


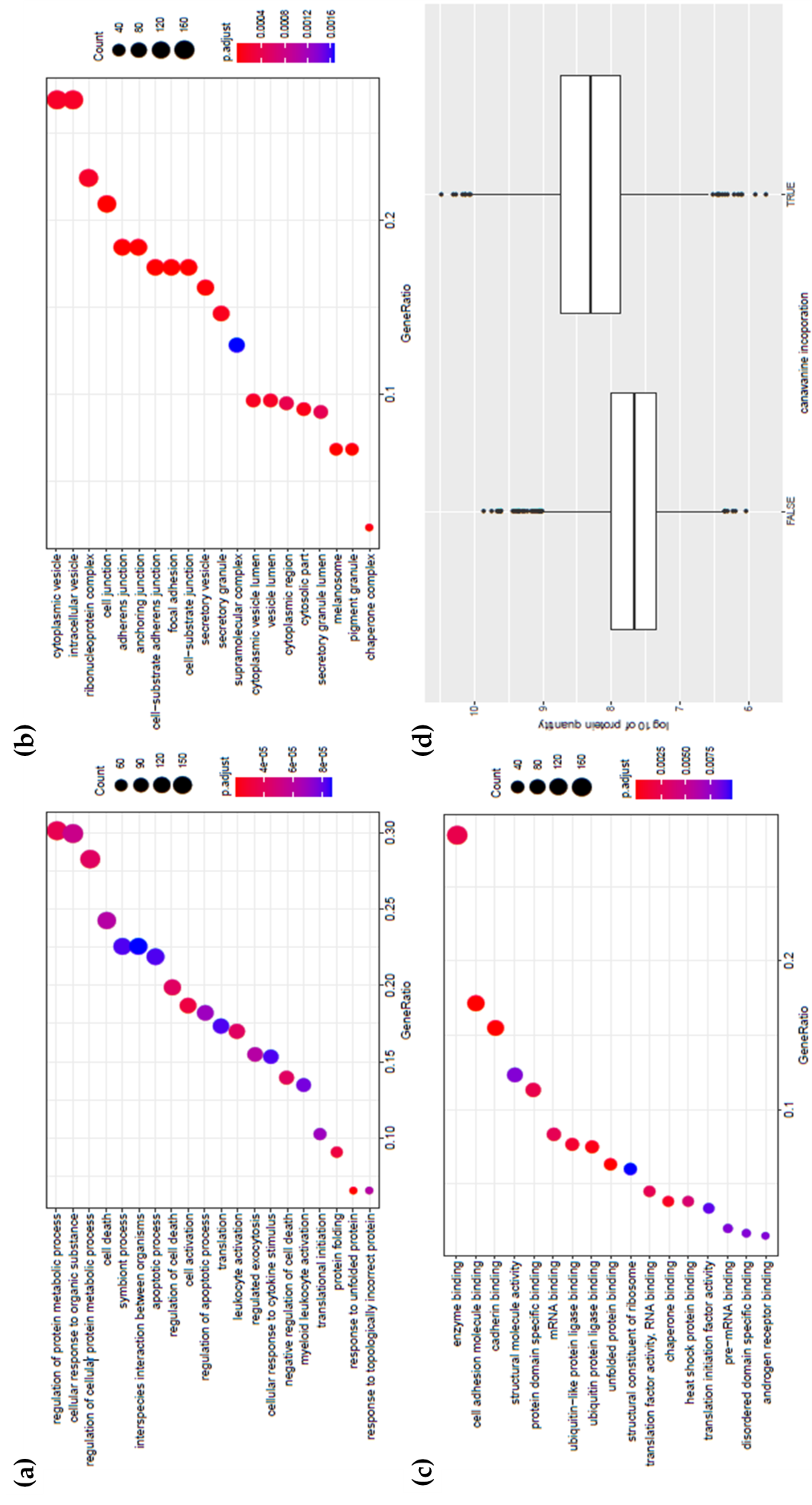


**Karatsai et al. Supplementary Figure S8**


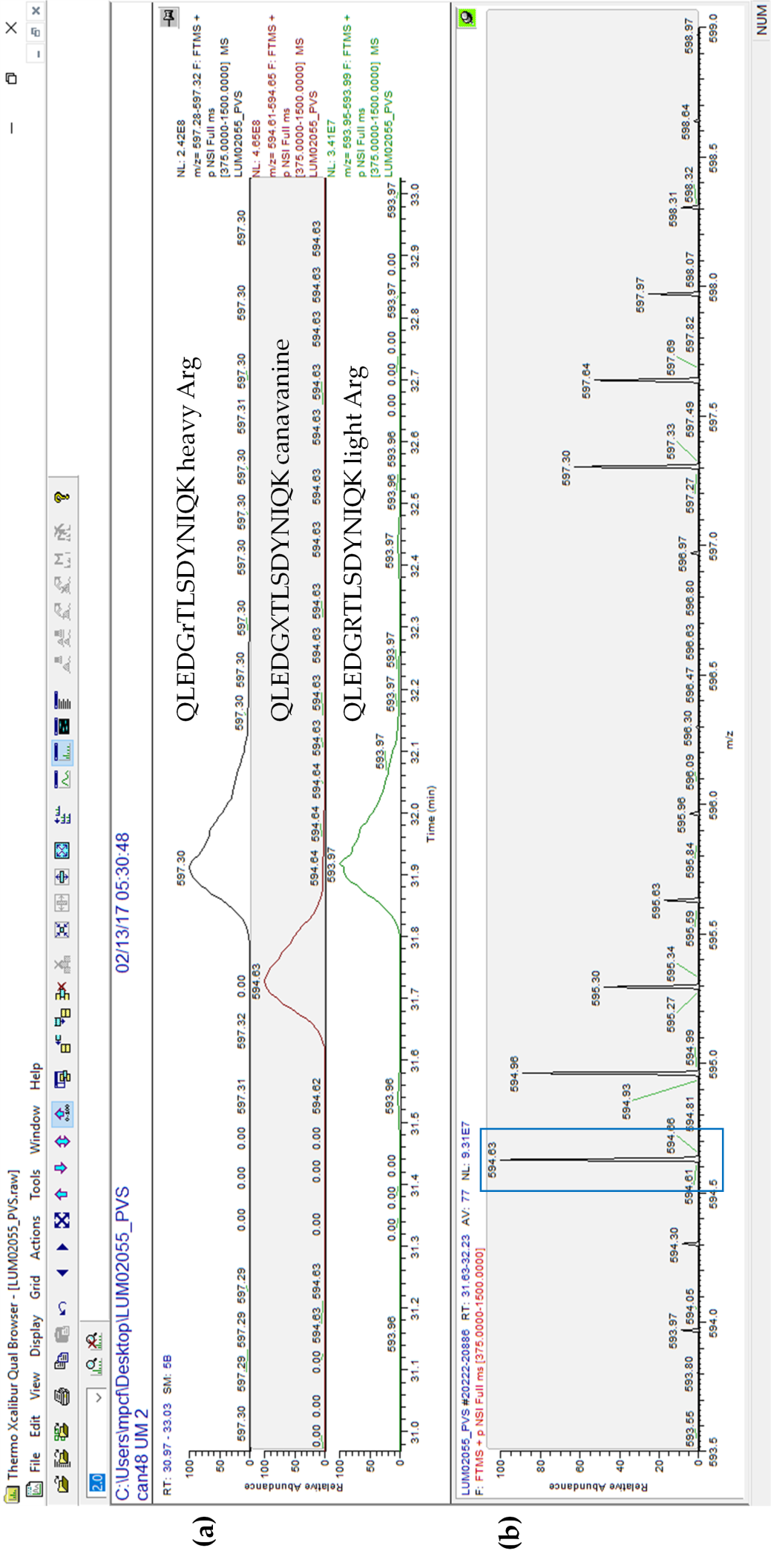


**Supplementary Figure S8.** Illustration of spectrum cohesion for QLEDGRTLSDYNIQK peptide. Three variants of the peptide were identified, where R designates light Arg; r – heavy Arg and X – canavanine. **(a)** Extracted chromatogrammes for the variants, **(b)** shows the 593.5-599 mass range (where the 3+ form is observed) of the integrated spectrum across the elution profile of all forms. Notably the 1st isotopic peak of canavanine containing variant and the 3rd peak of light Arg variant are not resolved (blue box).

**Karatsai et al. Supplementary Figure S9**

**
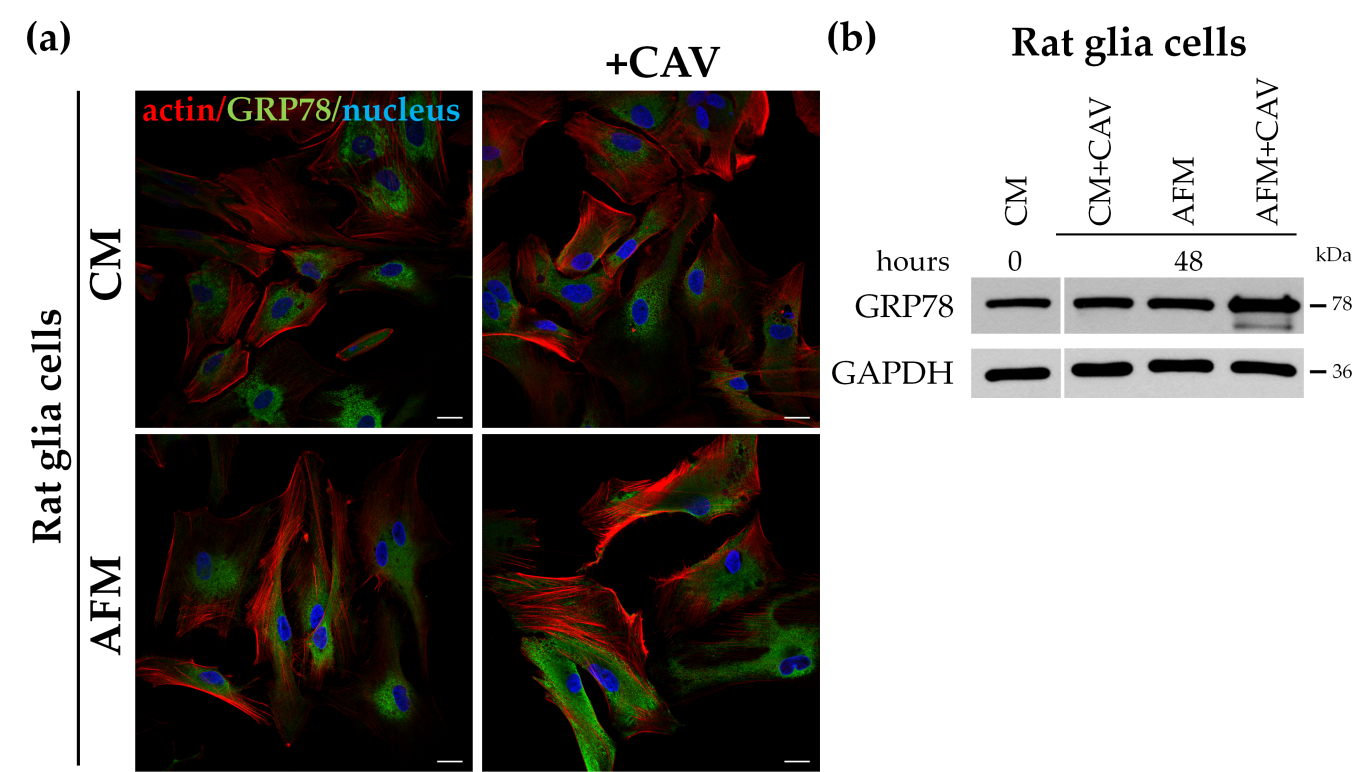
**

**Supplementary Figure S9.** Analysis of the ER stress marker, GRP78, in rat glia cells after 48 h of treatment with 50 µM canavanine under CM and AFM conditions. **(a)** Immunocytochemistry of GRP78, actin was labeled with Alexa Fluor 546-conjugated phalloidin. Nuclei were labeled with DAPI. Bars, 20 µm. **(b)** Detection of GRP78 in the rat glia cells lysates. GAPDH was used as a protein loading control.

**Karatsai et al. Supplementary Figure S10**

**
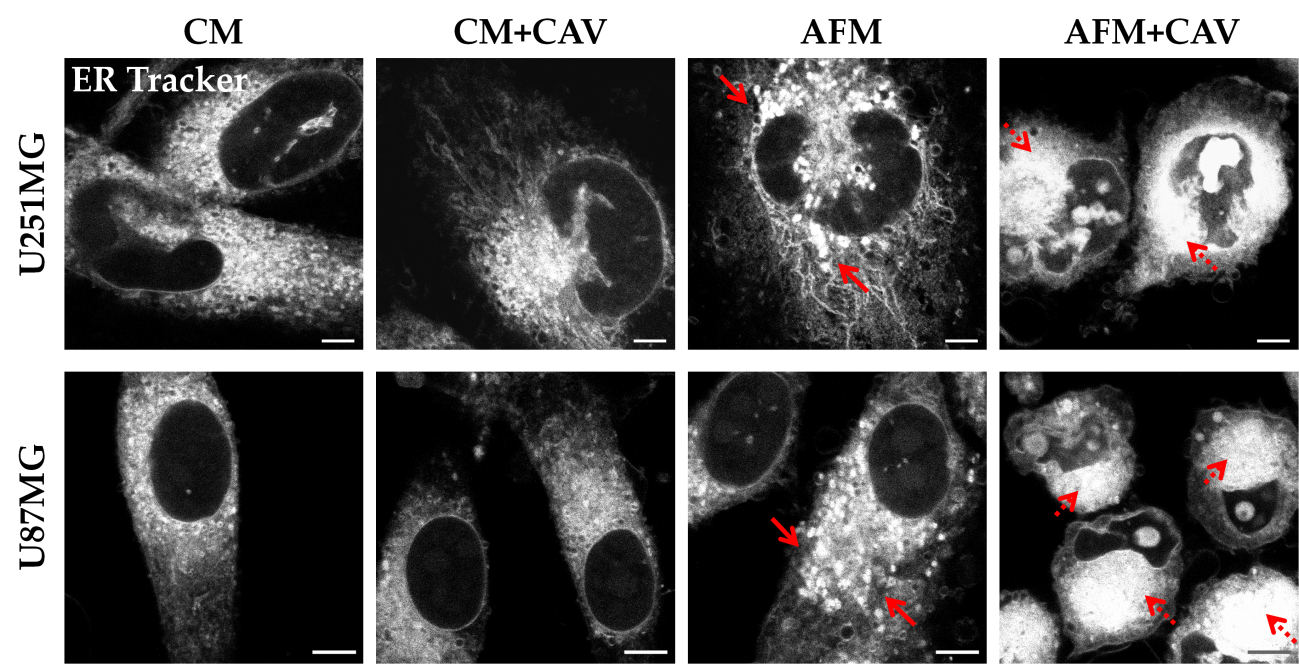
**

**Supplementary Figure S10.** Effect of 50 µM canavanine on the ER in U251MG and U87MG glioblastoma cell lines. Cells were treated for 48 h under CM or AFM conditions with or without canavanine. Later, cells were incubated with ER Tracker™ Blue/White DPX to visualize ER tubular network. Bars, 5 µm. Solid arrows indicate ER tracker-stained vesicles, and dotted arrows point to severely disintegrated ER.

**Karatsai et al. Supplementary Figure S11**

**
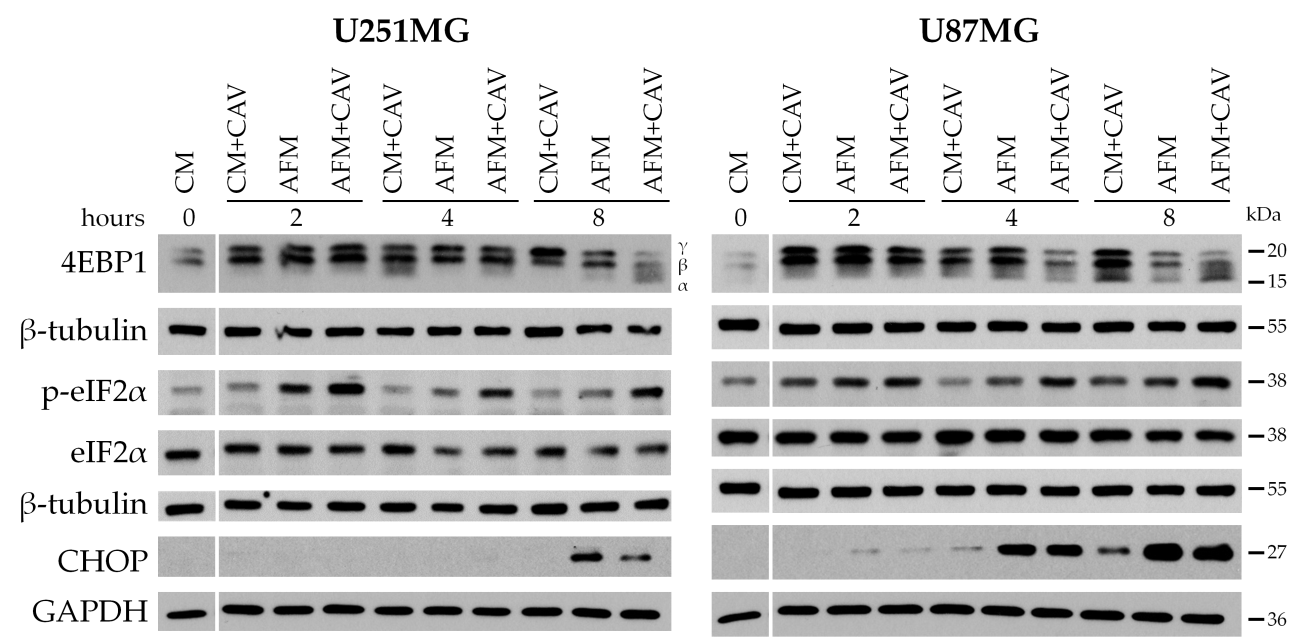
**

**Supplementary Figure S11.** Canavanine (50 µM) evokes early glioblastoma cell stress response already after 4 h of treatment under arginine deprivation. U251MG and U87MG cells were incubated for 2, 4, and 8 hours under experimental conditions. The analysis of cell lysates was made for the markers of the dynamics of protein synthesis (4EBP1, eIF2α and its phosphorylated form p-eIF2α) and proapoptotic marker (CHOP). GAPDH and β-tubulin were used as a protein loading controls.

**Karatsai et al. Supplementary Figure S12**

**
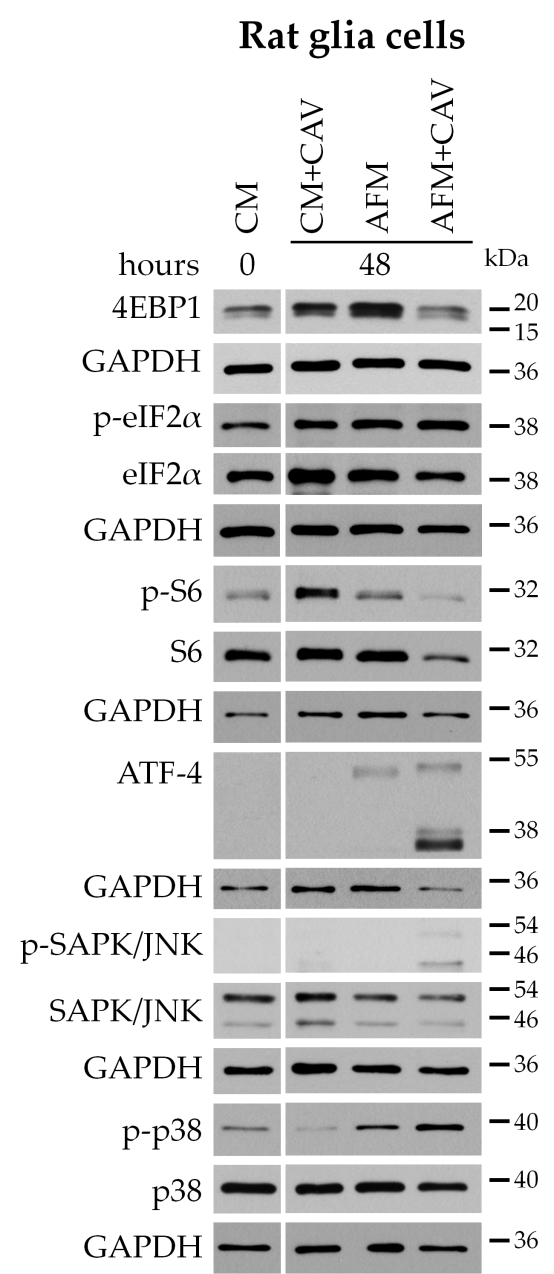
**

**Supplementary Figure S12.** Western Blot analysis of the markers of ER stress in rat glia cells treated with 50 µM canavanine under CM and AFM conditions. The analysis of cell lysates was performed for the markers of the dynamics of protein synthesis (4EBP1, eIF2α and S6) and stress markers (ATF-4, SAPK/JNK and p38) and their phosphorylated forms (p-eIF2α, p-S6, p-SAPK/JNK, and p-p38). GAPDH was used as a protein loading control.

**Karatsai et al. Supplementary Figure S13**


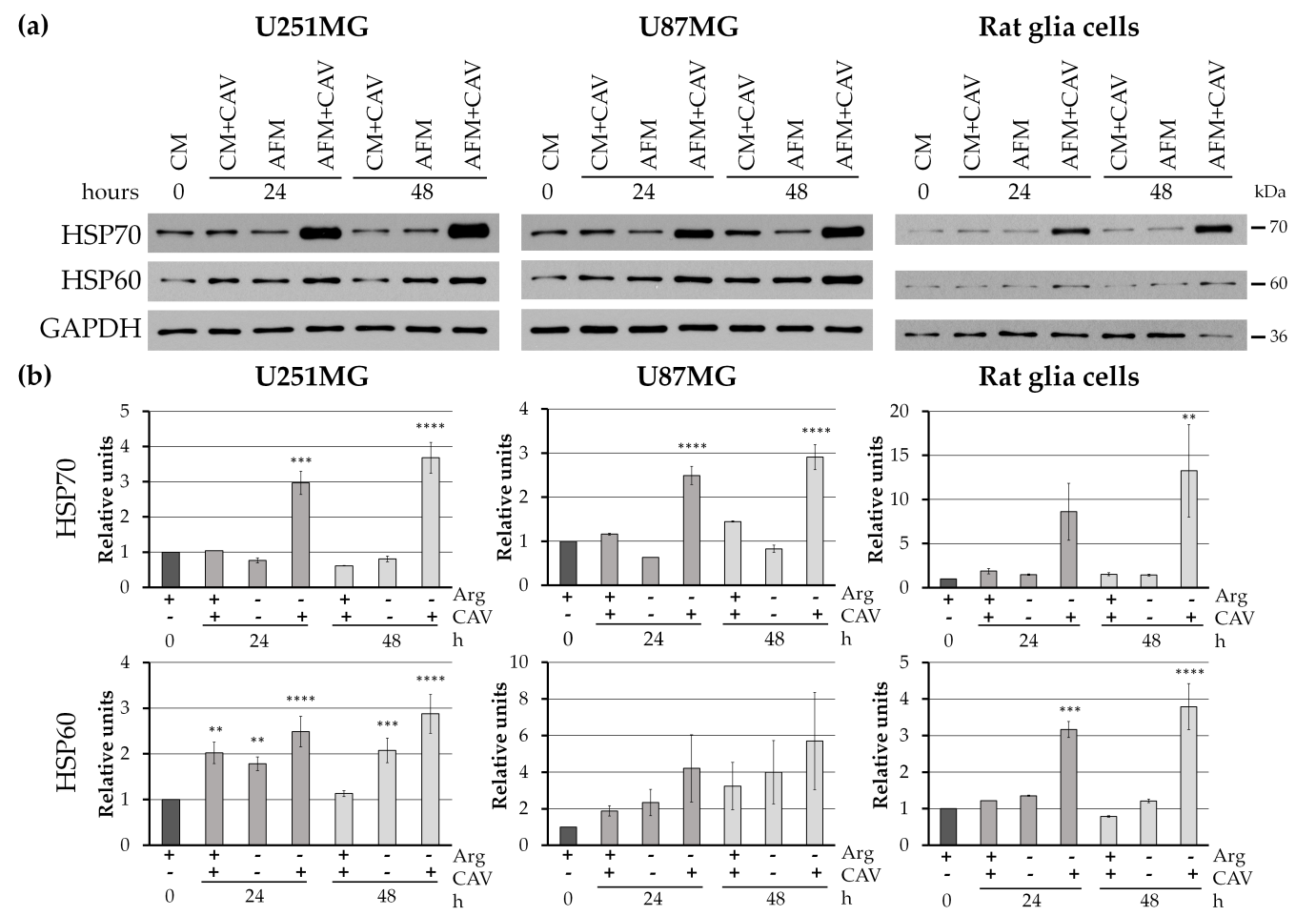


**Supplementary Figure S13.** The level of the heat shock proteins HSP70 and HSP60 as a markers of mitochondrial stress. U251MG, U87MG glioblastoma and normal rat glia cells were treated with 50 μM canavanine under CM and AFM conditions. **(a)** The levels of HSPs proteins were analyzed by Western Blot analysis. GAPDH was used as a protein loading control. **(b)** The densitometry of HSPs levels. Graph bars represent mean ±SD from three independent experiments. ** p<0.01, *** p<0.001, **** p<0.0001 relative to HSPs levels in cells cultured in CM (control, 1 relative unit).
